# TMEM166/EVA1A interacts with ATG16L1 and induces autophagosome formation and cell death

**DOI:** 10.1038/cddis.2016.230

**Published:** 2016-08-04

**Authors:** Jia Hu, Ge Li, Liujing Qu, Ning Li, Wei Liu, Dan xia, Beiqi Hongdu, Xin Lin, Chentong Xu, Yaxin Lou, Qihua He, Dalong Ma, Yingyu Chen

**Affiliations:** 1 Department of Immunology, Peking University School of Basic Medical Science; Key Laboratory of Medical Immunology, Ministry of Health, Peking University Health Sciences Center, 38 Xueyuan Road, Beijing 100191, China; 2 Research Center for Tissue Engineering and Regenerative Medicine, Wuhan Union Hospital, 1277 Jiefang Road, Wuhan 430022, China; 3 Department of Biochemistry and Molecular Biology, Program in Molecular and Cell Biology, Zhejiang University School of Medicine, 866 Yu-Hang-Tang Road, Hangzhou 310058, China; 4 Medical and Healthy Analytical Center, Peking University, 38 Xueyuan Road, Beijing 100191, China

## Abstract

The formation of the autophagosome is controlled by an orderly action of ATG proteins. However, how these proteins are recruited to autophagic membranes remain poorly clarified. In this study, we have provided a line of evidence confirming that EVA1A (eva-1 homolog A)/TMEM166 (transmembrane protein 166) is associated with autophagosomal membrane development. This notion is based on dotted EVA1A structures that colocalize with ZFYVE1, ATG9, LC3B, ATG16L1, ATG5, STX17, RAB7 and LAMP1, which represent different stages of the autophagic process. It is required for autophagosome formation as this phenotype was significantly decreased in *EVA1A-*silenced cells and *Eva1a* KO MEFs. EVA1A-induced autophagy is independent of the BECN1-PIK3C3 (phosphatidylinositol 3-kinase, catalytic subunit type 3) complex but requires ATG7 activity and the ATG12–ATG5/ATG16L1 complex. Here, we present a molecular mechanism by which EVA1A interacts with the WD repeats of ATG16L1 through its C-terminal and promotes ATG12–ATG5/ATG16L1 complex recruitment to the autophagic membrane and enhances the formation of the autophagosome. We also found that both autophagic and apoptotic mechanisms contributed to EVA1A-induced cell death while inhibition of autophagy and apoptosis attenuated EVA1A-induced cell death. Overall, these findings provide a comprehensive view to our understanding of the pathways involved in the role of EVA1A in autophagy and programmed cell death.

Autophagy is an evolutionarily conserved cellular process in which cytoplasmic components are sequestered in a double-membrane organelle known as the autophagosome and delivers them to the lysosome, leading to their breakdown.^1, 2^ More than 30 types of ATG proteins that participate in the formation of the autophagosome have been identified.^3^ The majority of these proteins are conserved from *S. cerevisiae* to other higher eukaryotes.^4^ Disorder of autophagy has been implicated in a wide range of diseases, including cancer, infections, autoimmunity and neurodegenerative diseases.

There are many factors that can stimulate autophagy, including nutrient starvation and energy deprivation. Upon starvation, the mTOR complex 1 (mTORC1) activates ULK1/Atg1 and BECN1-VPS34 complex activity, which are essential for PtdIns3P synthesis and omegasome formation. ZFYVE1, which binds PtdIns3P through its FYVE domains, is associated with the Golgi complex in normal cultured cells, translocates to an ER-associated omegasome upon starvation and is considered an omegasome marker. The ATG12–ATG5/ATG16L1 complex, LC3, ATG14 and WIPI2 have all been observed to be recruited to the omegasome, suggesting that the omegasome may function as a platform for autophagosome formation.^5^

It has been considered that the source of the autophagosomal membrane has multiple aspects, including the endoplasmic reticulum (ER), the Golgi apparatus, mitochondria, plasma membrane, recycling endosomes and ATG9-containing vesicles.^6, 7, 8, 9^ Although much progress has been made, a direct functional link between a membrane source and autophagosome biogenesis has not been established. Recently, Ge and coworkers developed a systematic membrane isolation scheme and defined the ER–Golgi intermediate compartment as a primary membrane determinant to trigger LC3 lipidation.^10, 11^ Graef *et al.*^12^ revealed that the ER exit sites (ERESs) (the specialized ER regions in which proteins are sorted into the secretory system) are key factors in the formation of autophagosomes. ERESs are the physical and functional core of the autophagosome biogenesis components.

Two ubiquitination-like processes regulate the elongation of isolation membranes. First, ATG12 is conjugated to ATG5 by ATG7, then the ATG12–ATG5 complex interacts with ATG16L1, and this resulting large molecular complex associates with IM.^13, 14, 15^ The second involves the conjugation of ubiquitin-like molecules of the LC3 to phosphatidylethanolamine (PE) by ATG7 and ATG3, resulting in autophagosome-associated LC3-II. The ATG12–ATG5/ATG16L1 complex may be able to enhance LC3 conjugation to PE by acting in an E3-like manner.^16, 17, 18^ In this way, the ATG12–ATG5/ATG16L1 complex may determine the sites of autophagosome synthesis by recruiting LC3 to the Atg12–Atg5-associated membranes. Although the ATG12–ATG5/ATG16L1 complex localizes to the IM or pre-phagophore structures, how the ATG12–ATG5/ATG16L1 complex reaches the PAS and regulates isolation membrane elongation remains largely unknown.

EVA1A (eva-1 homolog A), also known as TMEM166 (transmembrane protein 166) or FAM176A (family with sequence similarity 176), is a novel human gene involved in autophagy and apoptosis.^19, 20, 21^ A previous study showed that EVA1A is expressed in a cell- and tissue-specific manner. EVA1A expression is decreased in many types of human tumors, such as gastric cancer, esophagus cancer, adrenal cortical carcinoma, pituitary adenoma and parathyroid adenoma.^22, 23^ *In vivo* and *in vitro* experiments have demonstrated that EVA1A overexpression inhibits the proliferation of tumor cells and induces both autophagy and apoptosis even under nutrient-rich conditions, and the appearance of autophagy usually precedes cell death. Although we predict that EVA1A participates in regulating autophagy, the molecular mechanism by which this occurs has not been investigated.

In this paper, we found that EVA1A stimulates autophagy by interacting with WD repeats of ATG16L1. Furthermore, it acts on downstream of the BECN1 complex and upstream of ATG16L1 and may be responsible for ATG12–5/16L1 recruitment to the isolation membrane. EVA1A, potentially as a component of the autophagosomal membrane, is closely related to the development and maturation of the autophagosome. We also investigated the relationship between EVA1A-induced autophagy and cell death.

## Results

### EVA1A promotes autophagic flux

**Figure 1:**
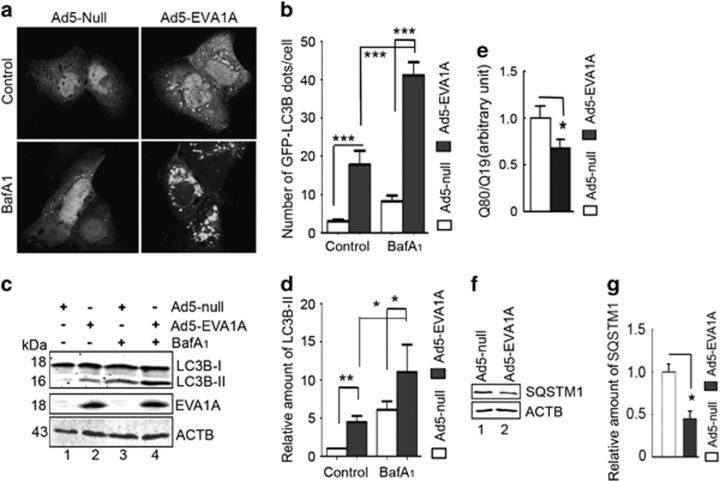
EVA1A overexpression promotes autophagic flux. (**a**) U2OS cells were infected with either Ad5-EVA1A or Ad5-null at 200 MOI combined with Ad5-GFP–LC3B at 50 MOI for 18 h, then treated with 10 nM BafA_1_ for the last 4 h. The distribution of GFP–LC3B was observed by confocal microscopy. (**b**) Quantification of GFP–LC3B puncta in cells treated with reagents as indicated in (**a**). Data are means±S.D. of at least 100 cells scored. (**c**) Western blot analysis of endogenous LC3B-II levels and EVA1A protein in U2OS cells treated as in (**a**). (**d**) Quantification of LC3B-II levels relative to ACTB in cells treated as in (**c**). The average value in Ad5-null infected cells without BafA_1_ treatment was normalized as 1. Data are means±S.D. of results from three experiments. (**e**) U2OS cells were transfected with polyQ80-luciferase (or control polyQ19-luciferase), then infected by Ad5-EVA1A (or Ad5-null) at 200 MOI for 18 h. Luciferase activities were monitored, and polyQ80-luciferase/polyQ19-luciferase ratios were calculated. (**f**) Western blot analysis of endogenous SQSTM1 levels in U2OS cells infected with either Ad5-EVA1A or Ad5-null at 200 MOI for 18 h. (**g**) Quantification of SQSTM1 levels relative to ACTB in cells treated as in (**f**). The average value in Ad5-null infected cells was normalized as 1. Data are means±S.D. of results from three experiments. *P<0.05, ** *P*<0.01, *** *P*<0.001

Previous studies have revealed that the overexpression of EVA1A has some features of autophagy under nutrient-rich conditions, such as the accumulation of LC3B-II and increased green fluorescent protein (GFP)–LC3B puncta. However, increased LC3B-II levels can be associated with either enhanced autophagosome synthesis or reduced autophagosome turnover.^24^ To discern the difference between them, we conducted our experiments in the absence or presence of vacuolar ATPase inhibitor bafilomycin A_1_ (BafA_1_), an inhibitor of the autophagic flux through raising lysosomal pH. Data from repeated experiments showed that Ad5-EVA1A significantly increased the occurrence of GFP–LC3B puncta when compared with Ad5-null transfected cells under nutrient-rich conditions, which was consistent with previous reports (Figures 1a and b, upper panel). Similarly, BafA_1_ treatment caused a further increase in GFP–LC3B dots in Ad5-EVA1A-infected cells (Figures 1a and b, lower panel). In line with these results, we next measured the endogenous LC3B conversion by western blot. We observed that Ad5-EVA1A obviously elevated the levels of endogenous LC3B-II with or without BafA_1_ treatment (Figures 1c and d, lane 2 *versus* lane 1, lane 4 *versus* lane 3). These data suggest that EVA1A promotes LC3B lipidation beyond the degradation blockade imposed by the inhibitor, indicating that the accumulation of LC3-II induced by the expression of EVA1A is unlikely to be attributable to the blockage of autophagic degradation.

We further examined the clearance of autophagic substrates in Ad5-EVA1A-infected U2OS cells using the accumulation of exogenously expressed polyQ80 aggregates as a surrogate marker for protein degradation.^24^ As shown in Figure 1e, compared with the Ad5-null, the accumulation of exogenous polyQ80 aggregates was downregulated in EVA1A-overexpressing cells. This treatment also resulted in a reduction of the endogenous autophagy substrate sequestosome 1 (SQSTM1) protein (Figures 1f and g).

**Figure 2:**
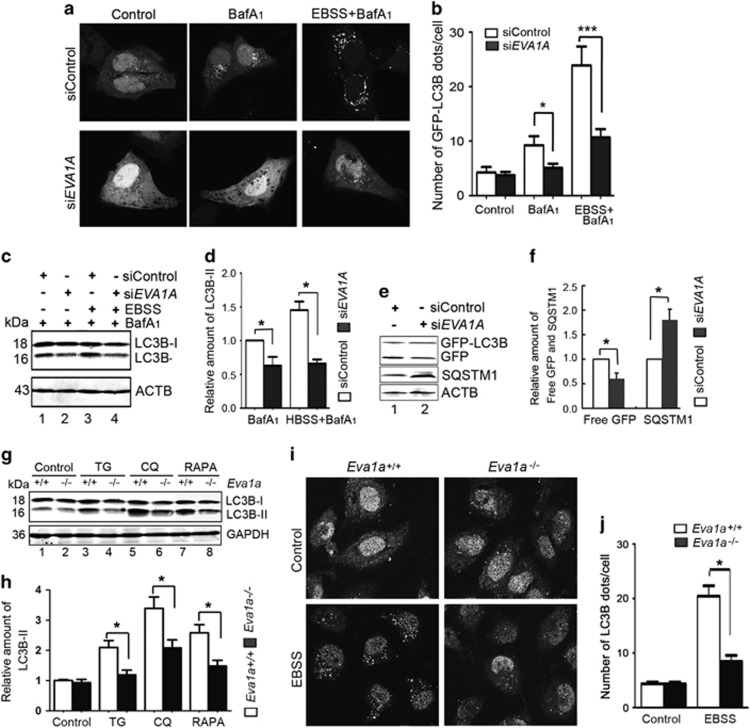
Knockdown of EVA1A impairs cell autophagy. (**a**) Representative confocal microscopy images of the GFP–LC3B distribution in U2OS cells transfected with *siControl* or *siEVA1A* for 48 h and treated with BafA_1_ (10 nM) and/or EBSS for the last 4 h. (**b**) Quantification of GFP–LC3B puncta in cells treated with reagents as indicated in (**a**). Data are means±S.D. of at least 100 cells scored. (**c**) Western blot analysis of endogenous LC3B-II levels in U2OS cells treated as in (**a**). (**d**) Quantification of amounts of LC3B-II relative to ACTB in cells treated as in (**c**). The average value in *siControl* transfected cells with BafA_1_ treatment was normalized as 1. Data are means±S.D. of results from three experiments. (**e**) HeLa cells stably expressing GFP–LC3B were transfected with *siControl* or *siEVA1A* for 48 h. Levels of free GFP and SQSTM1 were analyzed by western blot. (**f**) Quantification of the amount of free GFP or SQSTM1 relative to ACTB in cells treated as in (**e**). The average value in *siControl* transfected cells was normalized to 1. Data are means±S.D. of results from three experiments. (**g**) Western blot analysis of endogenous LC3B-II levels in MEFs treated as indicated. (**h**) Quantification of amounts of LC3B-II relative to ACTB in cells treated as in (**g**). The average value in Eva1a^+/+^ MEF cells without any treatment was normalized as 1. Data are means ± S.D. of results from three experiments. (**i**) Representative confocal microscopy images of endogenous LC3B distribution in MEFs treated with BafA_1_ (10 nM) and/or EBSS for 4 h. (**j**) Quantification of endogenous LC3B puncta in mef cells as indicated in (**i**). Data are means ± S.D. of at least 100 cells scored. *P<0.05, *** *P*<0.001

Further analysis was performed in *EVA1A*-silenced U2OS cells. We identified two effective small interfering RNAs (siRNAs) against EVA1A (*siEVA1A-2* and *siEVA1A-3*) via RT-PCR (Supplementary Figure S1a). Unless otherwise noted, *siEVA1A-2+3* hereafter will be designated simply as *siEVA1A* and the control siRNA as *siControl*. The results revealed that a knockdown of *EVA1A* reduced the puncta formation of GFP–LC3B by either BafA_1_ alone or together with Earle's balanced salt solution (EBSS), compared with the *siControl* (Figures 2a and b). The puncta distribution of endogenous LC3B was similar to that of GFP–LC3B in *EVA1A*-silenced U2OS cells (Supplementary Figures S1b and c). Next, we detected the steady-state level of endogenous LC3B-II protein by western blot. In a comparison of *siControl*-transfected cells, *siEVA1A* attenuated the accumulation of LC3B-II after treatment of BafA_1_ (Figures 2c and d, lane 2 *versus* lane 1). This reduction was maintained in the presence of EBSS (Figures 2c and d, lane 4 *versus* lane 3), indicating that the knockdown of *EVA1A* partly attenuated EBSS-induced LC3B lipidation.

Next, we measured functional autophagic flux. In GFP–LC3B stably expressing HeLa cells, we observed that the free GFP band in *EVA1A*-silenced cells was weaker than that in *siControl* cells (Figures 2e and f, upper panel). Simultaneously, the levels of SQSTM1 protein also increased (Figures 2e and f, lower panel), indicating that basic autophagic flux was impaired by the inhibition of EVA1A expression.

To further explore the biological activity of EVA1A in autophagy, *Eva1a* gene knockout (KO) mice were produced. In *Eva1a* KO mouse embryonic fibroblasts (MEFs), we observed that there was no obvious difference in the levels of LC3B-II between wild-type (WT) and *Eva1a* KO MEFs at the basic levels (Figures 2g and h, lane 2 *versus* lane 1). However, in the presence of chloroquine (CQ), the accumulation of LC3B-II was significantly reduced in *Eva1a* KO MEFs compared with the WT MEFs (Figures 2g and h, lane 6 *versus* lane 5), indicating that the deficiency in EVA1A attenuated the background level of autophagosome synthesis. Similarly, *Eva1a* KO MEFs also showed the decreased LC3B conversion triggered by TG or RAPA treatment (Figures 2g and h, lane 4 *versus* lane 3 and lane 8 *versus* lane 7). Data from the confocal analysis suggested that *Eva1a* KO MEFs were impaired LC3B puncta structures treated by EBSS compared with that *Eva1a* WT MEFs (Figures 2i and j). Collectively, these results indicated that EVA1A had a stimulative effect on autophagy under growing conditions.

### EVA1A associates with the autophagosomal membrane

The membrane origin of autophagosomes is one of the critical questions in the process of autophagy. It has been proposed that the autophagosome membrane originates from a number of sources, including the ER, the Golgi apparatus, mitochondria and the plasma membrane. We previously confirmed that EVA1A is an ER and lysosome-resident transmembrane protein in HeLa cells.^19^ Here, we also found that EVA1A partially exhibited *cis*-Golgi localization, because of an EVA1A colocalization with p58, not TGN46 (Supplementary Figures S2a and b). As correct localization and topology are crucial for the cellular function of a protein, we further used a fluorescence protease protection assay^25^ to determine which terminus of the EVA1A protein is lumenal and which one faces the cytosol. HEK293 cells were co-transfected with CFP and YFP-TMEM166-Cherry or Cherry-TMEM166-YFP and then treated with digitonin and trypsin. The fluorescent signals were recorded by live cell imaging. As shown in Supplementary Figures S2c and d, only the N-terminal YFP-EVA1A or Cherry-TMEM166 showed resistance to both cell permeabilization and protease treatment, which confirmed the topology of EVA1A as a type I membrane protein.

**Figure 3:**
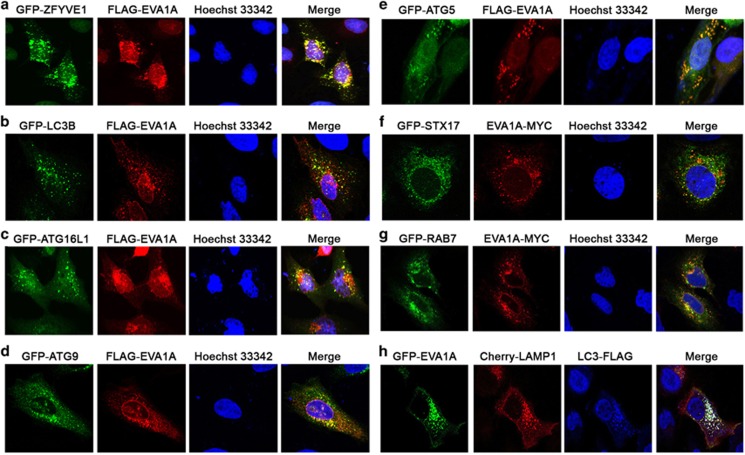
EVA1A colocalizes with the autophagosomal membrane. Confocal microscopy images are shown in U2OS cells: co-transfected with FLAG-EVA1A (or MYC-EVA1A) and GFP-ZFYVE1 (**a**), GFP–LC3B (**b**), GFP–ATG16L1 (**c**), GFP-ATG9 (**d**), GFP-ATG5 (**e**), GFP-STX17 (**f**) or GFP-RAB7 (**g**) and then immunostained with an anti-FLAG (or MYC) antibody after 24 h. Nuclei were stained with Hoechst 33342. (**h**) Co-transfected with GFP-EVA1A, Cherry-LAMP1 and LC3B-FLAG, and then immunostained with an anti-FLAG antibody after 24 h

Considering the localization of EVA1A in various subcellular organelles or vesicles, we questioned whether EVA1A was associated with autophagosomal membrane development and maturation. Therefore, we analyzed the colocalization of EVA1A with a series of key marker molecules in the kinetic autophagic process. It has been reported that ZFYVE1/DFCP1 redistributes from an ER/Golgi localization to specific sites on the ER (i.e., omegasomes) when autophagy is activated. Therefore, it is a marker of omegasomes. Phagophore nucleation at the omegasome is an early autophagic event linked to the recruitment of ATG9-marked membranes. ATG16L1 localizes to the autophagic isolation membrane at the beginning of elongation and dissociates from the membrane at the completion of autophagosome formation, whereas LC3B localizes to the elongating isolation membrane, autophagosomes, amphisomes and autolysosome. By confocal observations, we found that the FLAG-EVA1A signal accumulated in the punctate structures in the cytosol and colocalized with GFP-ZFYVE1/DFCP1 (Figure 3a), GFP–LC3B (Figure 3b), GFP–ATG16L1 (Figure 3c), GFP-ATG9 (Figure 3d) and GFP-ATG5 (Figure 3e). Data from live cell imaging also confirmed the colocalization of EVA1A and LC3B (data not shown), indicating that EVA1A may participate in the biogenesis and expansion of autophagosomal membranes.

It was confirmed that SNARE protein syntaxin17 (STX17) is recruited to completed autophagosomes, but not the incomplete autophagosome or phagophore. It interacts with cytosolic synaptosomal-associated protein 29 (SNAP-29) and lysosomal VAMP8 for autophagosome–lysosome fusion.^26, 27^ We explored the colocalization between STX17 and EVA1A. The confocal observation showed that dotted EVA1A structures colocalized with STX17 (Figure 3f), suggesting that EVA1A was present in the completed autophagosome.

Autophagosomes have been reported to fuse with early or late endosomes to form amphisomes, and then subsequently fuse with lysosomes to generate autolysosomes. Some proteins have been implicated in the autophagosome–endosome/lysosome fusion process, including lysosomal membrane proteins such as LAMP1, small GTPases (e.g., RAB7), and SNARE proteins.^28^ From Figure 3g, we observed that EVA1A-positive vesicles colocalized with RAB7. Simultaneously, it also colocalized with LC3B and LAMP1 (lysosomal marker). These data suggest that EVA1A remains spatially linked or tethered to autophagosomal structures during autophagosome maturation. Taken together, it has been proposed that EVA1A may be one of the components of the autophagosomal membrane, which associates with autophagic membranes at every stage of the autophagosomal biogenesis process. Moreover, the results further support that the ER and Golgi are the primary source of autophagosomal membranes.

### EVA1A-induced autophagosome formation is independent of the BECN1-PIK3C3 complex, associated with ATG12-5/16L1 complex

As mentioned above, knockdown of *EVA1A* could reduce the autophagy flux. To further investigate the step at which the autophagic process is interrupted in *EVA1A*-silenced cells, we performed a series of tests.

**Figure 4:**
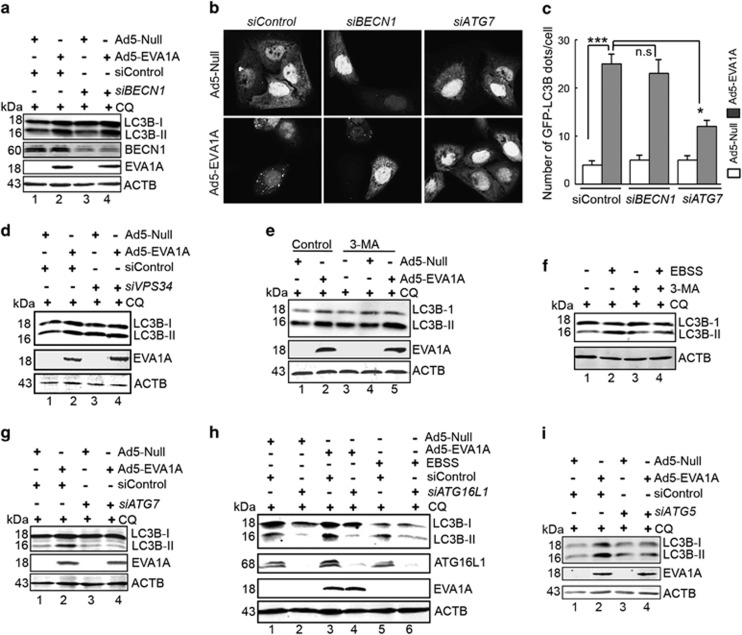
EVA1A-induced autophagosome formation is independent of the BECN1-PIK3C3 complex, associated with ATG12-5/16L1 complex. (**a**) U2OS cells were transfected with *siControl* or *siBECN1* for 24 h, infected with either Ad5-EVA1A or Ad5-null for 18 h and treated with CQ (25 mM) for the last 2 h. Western blot revealed the expression level of LC3B-II, BECN1 and EVA1A. (**b**) U2OS cells were transfected with *siControl* or *siBECN1 or siATG7* for 24 h, and infected with either Ad5-EVA1A or Ad5-null combined with Ad5-GFP–LC3B for 18 h. Representative confocal microscopy images are shown. (**c**) Quantification of GFP–LC3B puncta in cells treated with reagents as indicated in (**b**). Data are expressed as the mean±S.D. of at least 100 cells scored. (**d**) U2OS cells were treated with reagents as indicated, and the levels of LC3B-II and EVA1A were detected by western blot. (**e**) U2OS cells were infected with either Ad5-EVA1A or Ad5-null at 200 MOI for 18 h and then treated with CQ (25 mM) and/or 3-MA (10 nM) for the last 4 h. Western blot displayed the levels of LC3B-II and EVA1A. (**f**) U2OS cells were treated with the reagents as indicated, and the levels of LC3B-II were detected by western blot. (**g**) U2OS cells were transfected with *siControl* or *siATG7* for 24 h, infected with either Ad5-EVA1A or Ad5-null for 18 h, and treated with CQ for the last 2 h. The levels of LC3B-II and EVA1A were assessed by western blot. (**h**) U2OS cells were transfected with the *siControl* or *siATG16L1* for 24 h, infected with either Ad5-EVA1A or Ad5-null for 18 h, and treated with CQ (10 nM) and/or EBSS for the last 2 h. A western blot detected the levels of LC3B-II, ATG16L1, and EVA1A. (**i**) U2OS cells were treated with reagents as indicated, and the levels of LC3B-II were detected by western blot. *P<0.05, *** *P*<0.001, NS, not significant

A canonical mechanism of nucleation of autophagosomal precursors depends on BECN1/Beclin-1, which interacts with adaptor protein PIK3R4/p150 to stimulate the activity of phosphatidylinositol 3-kinase, catalytic subunit type 3 (PIK3C3)/VPS34. Therefore, we investigated if the BECN1-PIK3C3-dependent nucleation step could mediate EVA1A-induced autophagosome formation. U2OS cells were co-transfected with GFP–LC3B and *siBECN1* for 24 h, and then infected with either Ad5-Null or Ad5-EVA1A for 18 h. Surprisingly, *BECN-1* knockdown failed to prevent the increase in endogenous LC3B-II protein triggered by Ad5-EVA1A (Figure 4a, lane 4 *versus* lane 2). Furthermore, the deficiency of *BECN1* did not affect the number of GFP–LC3B puncta per cell induced by Ad5-EVA1A (Figures 4b and c, middle panel *versus* left panel), indicating a BECN1-independent mode of autophagy. Similarly, the LC3B lipidation induced by Ad5-EVA1A was not suppressed by *VPS34* knockdown (Figure 4d, lane 4 *versus* lane 2). Pharmacological inhibition by 3-methyladenine (3-MA), a PIK3C3 inhibitor did not prevent the LC3B conversion caused by Ad5-EVA1A (Figure 4e, lane 5 *versus* lane 2). As a control, 3-MA counteracted the levels of LC3B-II in EBSS-treated cells (Figure 4f, lane 4 *versus* lane 2). Collectively, these results suggest that EVA1A-triggered unconventional autophagy that bypassed the BECN1-PIK3C3 complex-dependent phagophore nucleation step.

The elongation of precursor membranes driven by ATG7, ATG5 and ATG16L1-mediated conjugation reactions represent a key step in the formation of autophagic vesicles. We next examined if these key autophagy-related proteins have a role in the induction of autophagy in EVA1A-overexpressed cells. U2OS cells were treated with siRNA against *ATG7*, and then infected with either Ad5-Null or Ad5-EVA1A. We found that the knockdown of *ATG7* depressed the occurrence GFP–LC3B puncta per cell induced by Ad5-EVA1A (Figures 4b and c, right panel *versus* left panel). Consistent with this finding, the conversion of LC3B-II caused by Ad5-EVA1A was also reduced in *ATG7*-silenced cells in the presence of CQ (Figure 4g, lane 4 *versus* lane 2). In *ATG16L1-*depleted U2OS cells, the level of LC3B-II protein were obviously decreased with and without EBSS treatment (Figure 4h, lane 2 *versus* lane 1, lane 6 *versus* lane 5). Similarly, the knockdown of *ATG16L1* also blocked the accumulation of LC3B-II protein evoked by Ad5-EVA1A (Figure 4h, lane 4 *versus* lane 2). Similar results were also observed in *ATG5* knockdown cells (Figure 4i). Combined with the results of Figure 3, in which EVA1A colocalized with ATG16L1, ATG5 and LC3B, these data indicated that EVA1A-mediated autophagy was dependent on the ATG7 activity and the ATG12-5/16L1 complex.

### Both autophagic and apoptotic mechanisms contributed to EVA1A-induced cell death

Accumulating evidence reveals that autophagy and apoptosis can cooperate, antagonize or assist each other, thus influencing the fate of the cell differentially. We have previously shown that EVA1A overexpression can induce cell death, which has dual characteristics of autophagy and apoptosis, with autophagy being preferentially induced.^19, 20, 21^ The crosstalk between apoptosis and autophagy is complex, as autophagy can function to promote cell survival or death under various experimental conditions. Therefore, we were interested in determining the possible functional interplay between autophagy and apoptosis in EVA1A-overexpressed cells.

**Figure 5:**
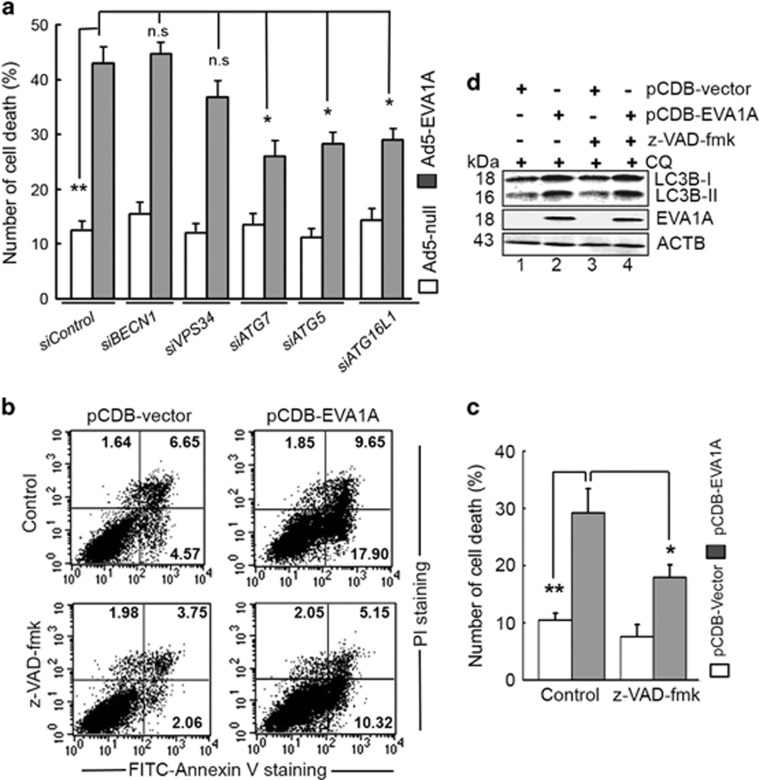
EVA1A-induced autophagy and apoptosis contributed to cell death. (**a**) U2OS cells were transfected with *siControl* or indicated siRNA against ATGs for 24 h, and were infected with either Ad5-EVA1A or Ad5-null for 24 h. Then, the cells were stained with FITC–Annexin V plus PI. Flow cytometry analyzed the level of cell death. Data are expressed as the mean±S.D. of the results from three separate experiments. (**b**)U2OS cells were treated with the control (0.01% DMSO) or z-VAD-fmk (40 *μ*M) for 2 h, then transfected with p-CDB-EVA1A, or vector for 24 h. The magnitude of cell death was measured by FITC–Annexin V and PI staining followed analysis by flow cytometry. (**c**) Cell treatment was as same as (**b**). Data are expressed as the mean±S.D. of the results from three independent experiments. (**d**) U2OS cells were incubated with or without z-VAD-fmk (40 *μ*M ) for 2 h, then transfected with p-CDB-EVA1A or vector for 18 h, and treated with CQ (10 nM) for the last 2 h. Effect of Z-VAD-FMK on the conversion of LC3B-I to LC3B-II in Ad5-EVA1A treated cells was analyzed by western blot. The western blot detected the levels of LC3B-II and EVA1A. *P<0.05, ** *P*<0.01, NS, not significant

U2OS cells were treated with siRNAs against core autophagy genes, such as *siBECN1, siATG16Ll*, *siVPS34*, *siATG7*, *siATG5* and *siControl* for 24 h, respectively. Then, cells were infected with either Ad5-Null or Ad5-EVA1A for 24 h and cell death was analyzed by flow cytometry. Surprisingly, the knockdown of *BECN1* and *VPS34* failed to prevent cell death, compared with the *siControl* treated cells (Figure 5a). However, the knockdown of *ATG7*, *ATG5* and *ATG16Ll* could partially block the cell death caused by Ad5-EVA1A (Figure 5a). Linked to the results of Figure 4, Ad5-EVA1A-induced autophagy was significantly impaired by the knockdown of *ATG7*, *ATG5* and *ATG16Ll*. Thus, the functional impact of the induction of autophagy in the context of Ad5-EVA1A cytotoxicity is of concern. These data suggested that Ad5-EVA1A could induce BECN1-independent autophagic cell death (ACD). Interestingly, autophagy stimulators, such as EBSS and rapamycin (RAPA) could enhance Ad5-EVA1A-induced cell death (Supplementary Figures S3a and b), implying that this autophagy inducer may act in concert with Ad5-EVA1A to induce ACD in this model. In addition, we also found that Ad5-EVA1A-infected U2OS cells in combination with chloroquine significantly increased the percentage of cell death (Supplementary Figure S3c), suggesting that blocking the degradation of the autophagosome may increase EVA1A-mediated cell death. However, we cannot rule out the possibility that chloroquine may also affect additional processes besides autophagy. Taken together, these results implied that the autophagy was required for EVA1A-triggered cell death.

We previously reported that EVA1A could increase the activities of caspase 9 and caspase 3 and induce cell apoptosis.^20^ Here, we found that pre-treatment with the pan-caspase inhibitor (z-VAD-fmk) partially abrogated cell death induced by EVA1A overexpression compared with the control vector (Figures 5b and c). However, z-VAD-fmk failed to inhibit EVA1A-mediated autophagy (Figure 5d), indicating that apoptosis was also responsible for cell death evoked by EVA1A. Collectively, these findings suggest that both autophagy and apoptosis are required in parallel pathways to contribute to cell death triggered by EVA1A overexpression.

### Structure–function correlation of EVA1A mutants

**Figure 6:**
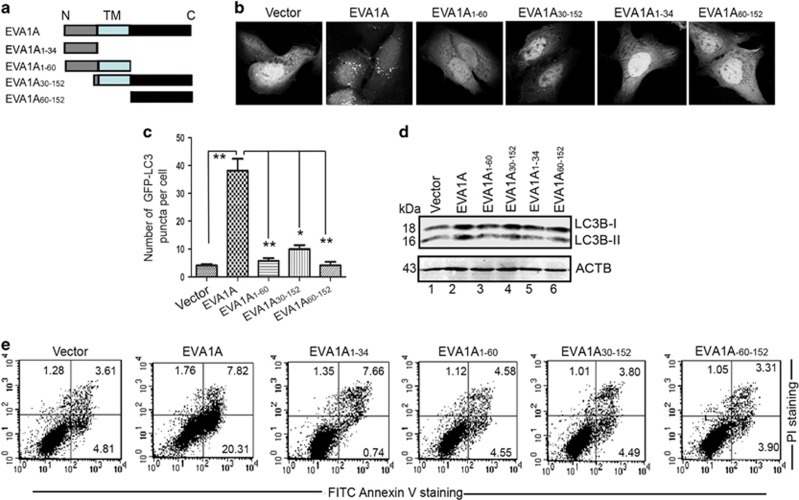
Structure–function correlation of EVA1A mutants. (**a**) Schematic representations of the WT EVA1A and its mutants. (**b**) Representative confocal microscopy images of GFP–LC3B distribution in U2OS cells transfected with indicated plasmids for 18 h. (**c**) Quantification of GFP–LC3B puncta per cell treated as in (**b**). Data are the mean±S.D. of at least 50 cells scored (*P<0.05, **P<0.01). (**d**) Western blot analysis of endogenous LC3B-II levels in U2OS cells treated as in (**b**). (**e**) U2OS cells were transfected with indicated plasmids for 36 h. Apoptotic cells were measured by FITC–Annexin V and PI staining followed by flow cytometry analysis. *P<0.05, ** *P*<0.01

EVA1A containing 152 amino-acid residues is a well-conserved protein, sharing significant homology to the corresponding proteins between species. To advance investigations into the structure–function correlation of EVA1A, several of the EVA1A mutants were generated (Figure 6a). First, we characterized the autophagic activities of these EVA1A mutants. Consistent with the above observation, WT EVA1A significantly induced punctuated GFP–LC3B formation (Figures 6b and c) and endogenous LC3B-II accumulation was comparable to the vector control (Figure 6d lane 2 *versus* lane 1). However, EVA1A mutants failed to display any autophagic phenotype (Figures 6b–d). We also investigated the effects of EVA1A mutants on cell death. Data from flow cytometry revealed that WT EVA1A markedly evoked cell death compared with vector-transfected cells (Figure 6e). These EVA1A mutants could not mediate cell death. Taken together, these findings suggest that intact EVA1A is necessary for its biological activities.

There is some relationship between function and the position of a protein. Here, we analyzed the cell location of these EVA1A mutants. The expression of FLAG-EVA1A_60-152_ was diffused throughout the cell cytoplasm and failed to colocalize with either GFP-ZFYVE1 (Supplementary Figure S4a) or GFP–LC3B (Supplementary Figure S4b). However, it was positioned in diffused GFP–ATG16L1 (Supplementary Figure S4c). The FLAG-EVA1A_30-152_ protein presented with a dot distribution in the cell cytoplasm and nucleus; it had no significant colocalization with GFP-ZFYVE1 (Supplementary Figure S4d), GFP–LC3B (Supplementary Figure S4e) or GFP–ATG16L1 (Supplementary Figure S4f). Similarly, FLAG-EVA1A_1-60_-positive puncta also failed to colocalize with GFP–ZFYVE1 (Supplementary Figure S4g), GFP–LC3B (Supplementary Figure S4h) and GFP–ATG16L1 (Supplementary Figure S4i). Despite the presence of TM domains, the localization of WT EVA1A and FLAG-EVA1A_30-152_ and FLAG-EVA1A_1-60_ in the cells was completely different (Figure 3 *versus* Supplementary Figures S4d–i), indicating a functional difference. These results suggest that the full-length EVA1A is required for its specific localization to the autophagosomal membrane and subsequent biological activity.

### EVA1A interacts with ATG16L1 via its C-terminal and ATG16L1 is required for EVA1A activity

**Figure 7:**
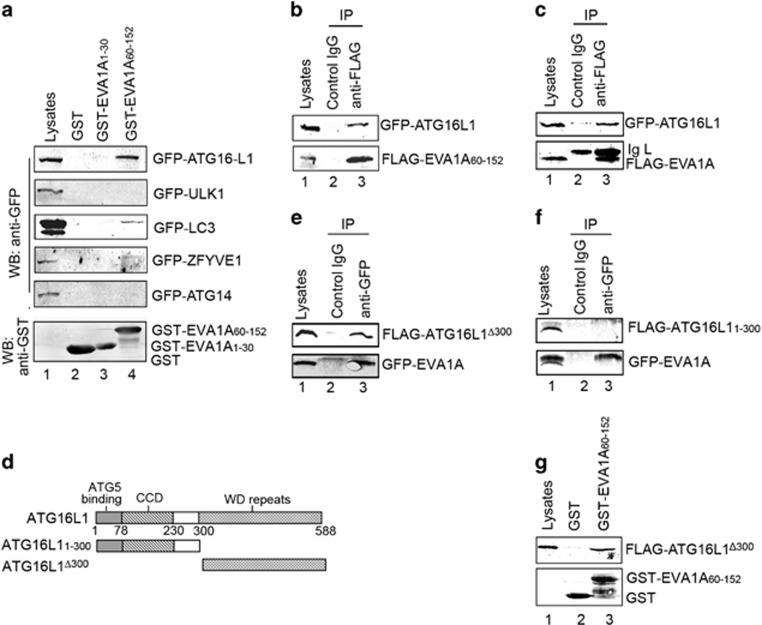
EVA1A is associated with ATG16L1 via its C-terminal. (**a**) GST-EVA1A_1–30_, the GST-EVA1A_60–152_ fusion protein, and the GST protein immobilized on glutathione-sepharose beads were incubated with HeLa cell lysates containing GFP-DFCP1, GFP-ULK1, GFP-LC3, GFP-ATG14 or GFP–ATG16L1, respectively. GFP and GST were detected in the washed beads by western blot. (**b** and **c**) HeLa cells were co-transfected with GFP–ATG16L1 and FLAG-EVA1A_60__–__152_ or FLAG-EVA1A for 24 h. Total cell extracts were subjected to IP using either an anti-FLAG or a nonspecific control mIgG as indicated; GFP and FLAG were detected in the washed beads by western blot. (**d**) Schematic representations of the WT ATG16L1 and its mutants. (**e** and **f**) HeLa cells were co-transfected with GFP-EVA1A and FLAG-ATG16L1^△300^ or FLAG-ATG16L1_1–300_ for 24 h. Total cell extracts were subjected to IP using either an anti-FLAG or a nonspecific control mIgG, as indicated. GFP and FLAG were detected in the washed beads by western blot. (**g**) GST-EVA1A_60–152_ fusion protein and the GST protein immobilized on glutathione-sepharose beads were incubated with FLAG-ATG16L1^△300^ transfected HeLa cell lysates at 4 °C for 4 h. GFP and GST were detected in the washed beads by western blot

To obtain mechanistic insight as to which molecular mediator was responsible for facilitating EVA1A-induced autophagy, we tested the interaction between EVA1A and several autophagy-related molecules. Initially, we sought to obtain a glutathione *S*-transferase (GST)-tagged full-length EVA1A protein and perform a pulldown assay. To our surprise, the cultured *Escherichia coli* died when adding IPTG to induce full-length GST-EVA1A expression. This phenotype may be due to an unknown EVA1A toxicity. Therefore, we constructed a GST-tagged EVA1A mutant lacking TM (GST-EVA1A_1__–30_ and GST-EVA1A_60__–152_) to complete the series of pulldown assays. As shown in Figures 7a, a strong association of GST-EVA1A_60__–152_ with GFP–ATG16L1 was detected, but not with GST or GST-EVA1A_1__–30_. GST-EVA1A_60__–152_ could also present weak binding with GFP-LC3. In addition, GST-EVA1A_60__–152_ failed to isolate GFP-ULK1, GFP-ZFYVE1 or GFP-ATG14.

To further confirm the correlation between EVA1A and ATG16L1, we performed a co-immunoprecipitation (co-IP) assay. FLAG-EVA1A_60__–52_ or full-length FLAG-EVA1A and GFP–ATG16L1 plasmids were co-transfected into HeLa cells. After 24 h, the cell lysates were subjected to IP with an anti-FLAG antibody. Consistently, western blot analysis revealed that both FLAG-EVA1A_60__–152_ and full-length FLAG-EVA1A coprecipitated with GFP–ATG16L1 (Figures 7b and c). FLAG-EVA1A_60_–_152_ could also present a weak binding with GFP-ATG5 (Supplementary Figure S5a).

The mammalian ATG16L1 protein contains an N-terminal ATG5-binding domain (residue 1–78), a coiled-coil domain (residue 79–230), and a C-terminal tryptophan-aspartic acid (WD)-repeat domain.^29^ The N-terminal ATG5-binding domain and the coiled-coil domain can mediate homo-multimerization and can interact with the ATG12–ATG5 conjugate. In addition, the WD repeats are protein interaction domains found in functionally diverse proteins, suggesting that there may be undiscovered binding partners of ATG16L1 that interact with this region. Therefore, we performed a deletion analysis to identify the EVA1A- binding domain of ATG16L1. Figure 7d is the ATG16L1 mutant constructs. Data from the co-IP analysis revealed that the FLAG-ATG16L1^Δ300^ (residues 1–300 deleted) could interact with GFP-EVA1A (Figure 7e), but FLAG-ATG16L1_1__–300_ (containing an ATG5-binding domain and a coiled-coil domain) was defective in binding to GFP-EVA1A (Figure 7f). This suggested that the C-terminal WD repeat domain of ATG16L1 was essential for the interaction with EVA1A. This region of ATG16L1 has previously been shown to be required for TMEM59 binding.^30^ To further substantiate our findings, we performed pulldown assay. Consistent with the results of the Co-IP assay, GST-EVA1A_60__–152_ indeed binds to FLAG-ATG16L1^Δ300^ directly (Figure 7g). Simultaneously, GST-EVA1A_60__–152_ could precipitate GFP–ATG16L1 from cell lysates. It could also present a weak binding with ATG12–ATG5 conjugates (Supplementary Figure S5b). Given the observed colocalization of EVA1A and ATG16L1 or ATG5 (Figures 3c and 3e), and knockdown of *ATG16L1* or *ATG5* suppressed EVA1A-induced cell autophagy and cell death (Figures 4h and I,Figure 5a), these results suggested that ATG12–5/16L1 complex was required for EVA1A-mediated biological activities.

### Knockdown of *EVA1A* decreases the colocalization between LC3B and ATG16L1, as well as ATG12–ATG5 conjugates

**Figure 8:**
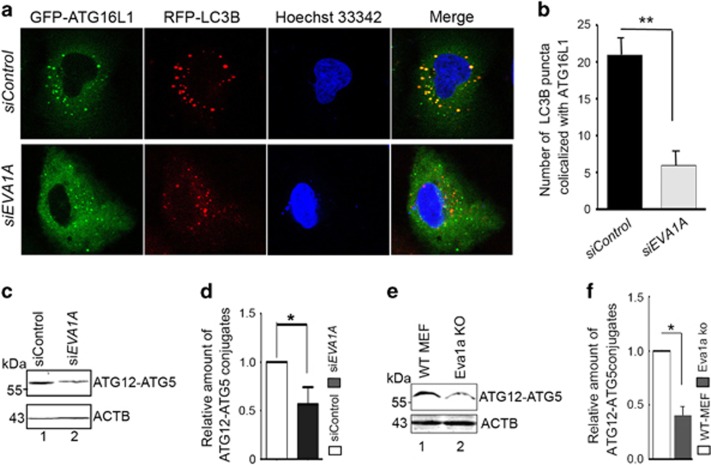
Knockdown of *EVA1A* decreases the colocalization between LC3B and ATG16L1. (**a**) U2OS cells were transfected with *siControl* or *siEVA1A* for 24 h, transfected with GFP–ATG16L1 and RFP-LC3B for 24 h, and treated with EBSS for the last 2 h. Representative confocal microscopy images were shown. (**b**) Cell treatment was as same as (**a**). The number of RFP-LC3B puncta that colocalize with GFP–ATG16L1 were analyzed. Data are the mean±S.D. of at least 50 cells scored. **P<0.01. (**c**) Western blot analysis of ATG12–ATG5 conjugates in U2OS cells transfected with *siControl* or *siEVA1A* for 48 h. (**d**) Quantification of the amount of ATG12–ATG5 conjugates relative to ACTB. The average value in the *siControl* treated cells was normalized to 1. Data are the mean±S.D. of results from three experiments.*P<0.05. (**e**) Western blot analysis of ATG12–ATG5 conjugates in MEFs treated as indicated. (**f**) Quantification of the amounts of ATG12–ATG5 conjugates relative to ACTB. The average value in WT MEFs was normalized to 1. Data are expressed as the means±S.D. of the results from three experiments. *P<0.05, ** *P*<0.01

We further assessed the role of the EVA1A–ATG16L1 association. During the process of IM elongation, LC3 is recruited to the ATG16L1-positive pre-autophagosomal structures, which ultimately form autophagosomes. We observed the colocalization between GFP–ATG16L1 and RFP-LC3 in *EVA1A*-depleted cells. As shown in Figures 8a and b, the knockdown of *EVA1A* decreased the colocalization of GFP–Atg16L1 with RFP-LC3 vesicles compared with the *siControl* transfected cells under conditions of starvation, indicating that the presence of ATG16L1 at autophagosomal membrane was at least partly dependent on EVA1A. Subsequently, we detected the level of ATG12–ATG5 conjugates and found that they were decreased in *EVA1A*-silenced cells (Figures 8c and d). Simultaneously, it was observed that Atg12–Atg5 conjugates were also downregulated in *Eva1a* KO MEFs compared with WT MEFs (Figures 8e and f). Taken together, it is possible to speculate that vesicle-located EVA1A may function as a membrane anchor for ATG16L1, participating or assisting in the homo-oligomerization of the ATG12−ATG5/ATG16L complex in the process of prolonging isolation membranes.

## Discussion

EVA1A, also termed TMEM166 or FAM176A, was first characterized in our lab.^19^ This gene is highly conserved in humans, chimpanzees, rats, mice and dogs, indicating its importance in vertebrate animals. The expression profile analysis indicates that the expression of the EVA1A protein in most cancer tissues is negative or lower compared with that of normal tissues.^22, 23^ The restoration of EVA1A in some cancer cell lines can induce cell death through both autophagy and apoptosis, suggesting that EVA1A is an effective tumor-suppressing molecule. However, the molecular mechanism by which EVA1A functions is unclear. Here, we have demonstrated that EVA1A, a vacuole-located type I membrane protein, has a stimulative effect on autophagic flux. It was shown to function downstream of the PIK3C3–BECN1 complex and upstream of ATG12−5/ATG16L1 complex. Evidence has been provided as follows: (1) siRNA-mediated *BECN1* downregulation, as well as *VPS34* silence, failed to inhibit the formation of autophagosome induced by EVA1A overexpression; (2) compounds that inhibit PtdIns3P production (e.g., 3-MA) inhibit autophagosome formation induced by starvation, but not EVA1A; (3) knockdown of *ATG7*, *ATG5* or *ATG16L1* significantly attenuates EVA1A-induced autophagosome formation. Further investigation indicated that EVA1A interacts with ATG16L1, and may be a membrane anchor, assisting ATG12−5/ATG16L1 complexes recruited to the autophagosome formation site or the autophagosomal membrane. In this manner, it could regulate and stably incorporate into the autophagosome. Similarly, ATG16L1 has an important role for EVA1A-mediated biological activities (Figures 4h and 5a).

To characterize the active domain in EVA1A, we constructed several mutants of EVA1A and found that only full-length EVA1A has the strongest activity. From our repeated observations, we found that EVA1A expression seems to be involved in the entire process of autophagosome membrane development and maturation. It associated with the omegasome, IM, autophagosome and autolysosome. This assessment is primarily derived from the observation that EVA1A-labeled punctate structures were positive for ZFYVE1, ATG9, ATG16L1, ATG5, LC3B, STX17, RAB7 and LAMP1. Data from live cell imaging also suggested that there is an almost complete colocalization between EVA1A and LC3B in autophagic cells. As EVA1A also interacts with the ER and the *cis*-Golgi apparatus, it is speculated that EVA1A prominently accumulates at the Golgi or ER, decorates autophagosomal membranes that resemble phagophores and autophagosomes, and remains during the fusion of autophagosomes with endosomes or lysosomes.

ATG9 is the only transmembrane protein, which is required for the autophagosome formation both in yeast and mammals. As the amount of lipids provided by a few ATG9 vesicles is not enough to support a complete autophagosome, other lipid sources are likely to exist. As EVA1A colocalizes with the ATG9 vesicle, the transmembrane protein EVA1A may be another source of lipid support, to assist or collaborate with ATG9. The two proteins have different aspects; Atg9 may transiently interact with isolation membranes and autophagosomes, and it may not be stably incorporated into autophagosomal membranes.^31^ In contrast, EVA1A is stably incorporated into autophagosomal membranes and remains until autophagosome maturation (Figure 3). Hence, the dots distribution and localization of EVA1A at ER-associated omegasomes, phagophores and autophagosomes, may also be a useful molecular marker for fluorescence-based EVA1A detection and analysis. At the same time, our findings may further provide important clues that ER and Golgi-derived vesicles/membranes are the membrane source of autophagosomes.

Accumulating literature confirmed that ATG16L1 can interact with a variety of molecules. ATG5 interacts with N-terminal of ATG16L1 (1–78AA), and FIP200 interacts with the middle region of Atg16L1 (229–242AA).^32^ Moreover, the ATG12–ATG5/ATG16L1 complex interacts with the ULK1-FIP200 complex to form a large complex in the cytoplasm, and is targeted to the autophagosome membrane.^33^ The wipi2b-binding site is located in ATG16L1 between 207 and 230. It is a PtdIns(3)P effector and is required for LC3 conjugation and starvation-induced autophagy through the recruitment of the ATG12–ATG5/ATG16L1 complex.^25^ It is also required for autophagic clearance of pathogenic bacteria. WIPI2 and FIP200 bind in adjacent, but distinct, regions of ATG16L1. NODs are also known to interact and recruit ATG16L1 to the bacterial entry site through an identified motif and therefore, act as ATG16L1 adaptor molecules enhancing bacteria-induced autophagy.^34^In this study, we identified a newly ATG16L1-binding partner; EVA1A interacts with the WD repeats of ATG16L1, which are absent in yeast Atg16. In fact, the function of C-terminal WD repeats in ATG16L1 is largely unknown. A recent report suggested that TMEM59 could bind to the WD repeat of ATG16L1, drive the local activation of LC3, and promote autophagy.^29^ Therefore, the role of both TMEM59 and EVA1A proteins is similar. Thus, these WD-domain binding proteins may be a receptor for the ATG12–ATG5/ATG16L1 complex on the isolation membrane. We propose that the WD repeats may provide a docking platform for protein adaptors that can engage ATG16L1 or the ATG12–ATG5/ATG16L1 complex. This interaction would leave the N-terminal part of the molecule free to interact with ATG5 and form the functional complex that drives LC3 lipidation.^28^ These studies may help to answer the suspending problem of what the function of this region may be.^28, 35^ However, more evidence is required to clarify this interaction.

The link between autophagy and cell death is complex and can be contradictory, however, it is critical to the fate of the cell. We are eager to elucidate the relationship between Ad5-EVA1A-induced autophagy and cell death. We used a siRNA against *Atg7* to genetically inhibit autophagy. Atg7 is an ubiquitin (E1)-like activating an enzyme that is critical to the modification of Atg12–Atg5/Atg16L1 complex and Atg8-PE (LC3-II in mammals), two important steps during autophagosome elongation and sequestration. We found that silencing *Atg7* reduced Ad5-EVA1A-induced autophagy and cell death. The same result was also found in *ATG5* and *ATG16L1*-silenced cell lines. The BECN1–VPS34 complex also has an important role in autophagy. However, we demonstrated that Ad5-EVA1A-induced autophagy is BECN1 independent, and the knockdown of BECN1 did not reduce Ad5-EVA1A-induced autophagy and cell death. Several studies have also revealed that autophagy can occur in a BECN1-independent manner.^36, 37, 38, 39^

To further explore the biological activity of EVA1A in autophagy, *Eva1a* gene KO mice were produced. A comparison of WT mice, *Eva1a* KO mice displayed normal vital signs, but no obvious phenotypic changes. Data from the studies of MEFs indicates that *Eva1a*^−/−^ MEFs still have a low level of autophagy. This phenotype is different from that of *Atg5*^−/−^ MEFs. In *Atg5*^−/−^ MEFs, the autophagy signals are completed disappeared both in basal or stress condition.^24^ This implies that the physiological changes caused by the *Eva1a* deletion may be filled by another compensatory mechanism. This also shows that the autophagy signaling mediated by EVA1A may be an auxiliary function, and not absolutely required.

As many anticancer drugs can engage autophagy, there are now a number of examples showing that the induction of ACD indeed represents a crucial event for the drug's antitumor activity. This opens new avenues for the development of novel therapeutic strategies and drug discovery. In addition, the engagement of ACD may offer new options to overcome treatment resistance, as autophagy has been reported to serve as a backup mechanism with important implications to bypass resistance, especially in apoptosis-refractory tumors. Repeated experiments indicate that the combination of EVA1A and the autophagy inducers, RAPA or EBSS, can significantly enhance cell death, suggesting that increased autophagy is a benefit for the antitumor efficacy of EVA1A. Given the known role of RAPA as an inhibitor of the MTOR signaling pathway, EVA1A may potentiate this effect.^20^ The dual inhibition of mTOR signaling may be an effective therapeutic strategy for cancer chemoprevention. Our investigation also found that chloroquine treatment also improved the EVA1A-induced cell death. Chloroquine blocks the degradation of autophagosomes, next to the much accumulation of proteins or organelles, which affect cell survival. It can be expected that there is the potential that EVA1A could be combined with chemotherapy in the treatment of tumors, which needs further exploration.

## Materials and Methods

### Plasmid construction, siRNA and adenoviral vectors

pCDNA3.1-EVA1A, GFP-EVA1A, FLAG-EVA1A, EVA1A-MYC and GST-EVA1A were generated in our laboratory. The following plasmids expressing truncated EVA1A were also constructed: EVA1A_1__–34_, EVA1A_1__–60_, EVA1A_60__–152_, EVA1A_30__–152_ (Figure 7a), GST-EVA1A_1__–34_, GST-EVA1A_60__–152_, FLAG-EVA1A_1__–60_, FLAG-EVA1A_60__–152_ and FLAG-EVA1A_30__–152_. GFP-ZFYVE1/DFCP1, RFP-LC3B, LC3B-FLAG, GFP-ATG9 and Cherry-LAMP1 were also generated. All plasmids were confirmed by DNA sequencing.

The polyQ80 and polyQ19-Firefly luciferase plasmids were kindly provided by Dr. Conrad C Weihl (Washington University School of Medicine, St. Loius, MO, USA). The GFP–LC3B plasmid was kindly provided by Dr. Zhenyu Yue (Mount Sinai School of Medicine, New York, NY, USA). GFP-ATG14, FLAG-ATG16L1, FLAG-ATG16L1_1__–300_ and FLAG-ATG16L1_300__–522_ were a gift from Dr. Noboru Mizushima (The University of Tokyo, Tokyo, Japan). GFP-Rab7 and GFP-STX17 were a gift from Dr. Hong Zhang (Chinese Academy of Sciences, Beijing, China). GFP-WIPI2 was a gift from Dr. Tassula Proikas-Cezanne (Eberhard Karls University Tuebingen, Tuebingen, Germany). Ad5-null and Ad5-EVA1A were purchased from SinoGenoMax (Beijing, China) as described previously.^20^

Double-stranded siRNAs against targeting sequences were designed, chemically synthesized by Genechem Corporation (Shanghai, China) (Supplementary Table s1). The control siRNA (siControl) was confirmed to have no matches with the complete human genome by a BLAST search in NCBI (www.ncbi.nlm.nih.gov).

### Cell culture, transfections and treatments

U2OS, HEK293 and HCT116 were cultured in DMEM (Invitrogen, Carlsbad, CA, USA, 12800-017) supplemented with 10% fetal bovine serum (FBS) and maintained at 37 °C in a humidified chamber with 5% CO_2_. GFP-LC3 stably expressing HeLa cell line was a gift from Dr. Li Yu (Tsinghua University, Beijing, China). Cells were transfected with plasmids using MegaTran 1.0 Transfection Reagent (ORIGEN, Rockville, MD, USA, TT200004) according to the manufacturer's instruction. The transfection of siRNA was performed by using Lipofectamine 2000 reagent (Invitrogen, Carlsbad, CA, USA, 11668-019). Autophagy was induced by nutrient deprivation through incubation in EBSS (contains neither amino acids nor FBS) or RAPA (1 *μ*M). Autophagy inhibition was achieved by treating cells with 3-MA (10 mM), BafA_1_ (100 nM) or CQ (25 mM), which can block autophagosome formation (3-MA) or the fusion of autophagosomes and lysosomes (BafA_1_ and CQ). Apoptosis inhibition was performed by treating cells with z-VAD-fmk (40 *μ*M), a pan-caspase inhibitor.

MEF primary cells were prepared from E15.5 embryos, cultured in DMEM supplemented with 10% FCS, and utilized for experiments between the second and the seventh passages.

### Reverse transcription PCR

Total RNA samples were extracted from cells with the TRIzol reagent (Invitrogen, 15596-026). RT-PCR was performed using the ThermoScript RT-PCR System (Invitrogen, 11146-024). Primers used for amplifying *EVA1A* were 5′-TGTCCCATGAGGCTGCCC-3′ (forward) and 5′-TCCCTAATAGTAGCGATTTCAGGCTC-3′ (reverse). GAPDH were 5′-GAAGGTGAAGGTCGGAGTC-3′ (forward) and 5′-GAAGATGGTGATGGGATTTC-3′ (reverse).

### Immunofluorescence, fluorescence and confocal microscopy

U2OS cells were cultured in confocal dishes and treated as indicated, fixed with 4% paraformaldehyde and permeabilized with 0.2% Triton X-100 (Beyotime, Shanghai, China, ST795). The dishes were then incubated with FBS overnight and exposed to primary antibody for 1 h at 4 °C. After being washed three times with phosphate-buffered saline (Solarbio, Beijing, China, P1010), the dishes were immersed with FITC/RBITC-conjugated secondary antibodies against mouse (bs-0296G-FITC/bs-0296G-RBITC, Bioss Inc., Woburn, MA, USA) or rabbit (bs-0295G-FITC/bs-0295G-RBITC, Bioss Inc.,). Nuclei were stained with Hoechst 33342. Morphological alterations in the cells were observed and documented with an Olympus FluoView FV1000 Confocal Microscope (Olympus, Melville, NY, USA). To observe the endogenous LC3B puncta, cells were fixed with 4% paraformaldehyde, permeabilized with 0.2% Triton X-100, incubated with FBS overnight, exposed to LC3B antibody, stained with an FITC-conjugated secondary antibody, and then observed by confocal microscopy. Cells transfected with GFP-LC3 or GFP-ZFYVE1 plasmids were observed by fluorescence microscopy. The number of LC3B puncta per cell or the percentage of enlarged GFP-ZFYVE1 structures was assessed in 10 non-overlapping fields, and statistical data were obtained from three independent experiments.

### Poly Q degradation assay

U2OS cells were co-transfected with the polyQ80-luciferase or control polyQ19-luciferase and the indicated plasmids using MegaTran 1.0 Transfection Reagent (Rockville, MD, USA) according to the manufacturer's protocol. After treatment for the desired time, cell lysates prepared from polyQ80-luciferase or polyQ19-luciferase transfected cells were dispensed in triplicate into a 96-well assay plate (COSTAR, Corning Inc., Corning, NY, USA 3925) containing 100 ml of Luciferase Assay Buffer II (containing luciferase assay substrate) from the DLRTM Assay System according to the manufacturer's protocol. Then the assay plate was mixed by pipetting two or three times, and the stabilized luminescent signal was measured by the Veritas Microplate Luminometer (Turner Biosystems, Sunnyvale, CA, USA). Data were expressed as the ratio of polyQ80-luciferase/polyQ19-luciferase luminescence signal values in each group as described previously.^40, 41^ All samples were assayed in triplicate, and the results were shown with three independent experiments.

### Flow cytometry analysis

Treated cells were trypsinized, washed with PBS and resuspended in 100 *μ*l binding buffer (10 mM HEPES, pH 7.4, 140 mM NaCl, 1 mM MgCl_2_, 5 mM KCl, 2.5 mM CaCl_2_). FITC-conjugated Annexin V was added to a final concentration of 0.5 *μ*g/ml. After incubation for 30 min at room temperature in the dark, propidium iodide (PI) was added to 1 *μ*g/ml, and the samples were immediately analyzed on a FACSCalibur flow cytometer (Becton Dickinson, Franklin lake, NJ, USA).

### IP and western blot

For the IP analysis, treated cells were collected and disrupted in RIPA Lysis Buffer containing protease inhibitors (Roche Diagnostics, Berlin, Germany, 04693116001). Total cell extracts (1 mg per sample) were mixed with precleared protein G sepharose^TM^ Fast Flow (GE Healthcare, Glattbrugg, Switzerland, 17-0618-01) and appropriate antibodies, followed by incubation for 4 h at 4 °C. The beads were collected by centrifugation, washed five times using washing buffer, resuspended in 2 × SDS loading buffer and then analyzed by western blot as described previously.^41^The protein bands were visualized using DyLight 800/DyLight 680-conjugated secondary antibodies, and the infrared fluorescence image was obtained using an Odyssey infrared imaging system (LI-CORBiosciences, Lincoln, NE, USA).

### GST affinity isolation assay

Recombinant GST or GST-EVA1A_60__–152_ or GST-EVA1A_1__–30_ fusion proteins were expressed in *E. coli* strain BL21 (DE3) and purified. Equal amounts of these proteins were mixed with whole-cell lysates extracted from plasmid transfected cells and glutathione-Sepharose 4B (GE Healthcare, 17-0756-01) for 4 h at 4 °C. After five washes, the beads were resuspended in 2 × SDS loading buffer and analyzed by western blot.^41^

### Statistical analysis

Results are presented as the mean±S.D. Differences between groups were analyzed using the Student's *t*-test for continuous variables. Statistical significance in this study was set at *P*<0.05. All reported *P*-values are two sided. All analyzes were performed with GraphPad Prism 5.

## Abbreviations

3-MA: 3-methyladenine
ACD: autophagic cell death
ATG: autophagy-related
BafA_1_: bafilomycin A_1_
BECN1: beclin-1
CQ: chloroquine
EBSS: Earle's balanced salt solution
EVA1A: eva-1 homolog A
FBS: fetal bovine serum
GFP: green fluorescent protein
GST: glutathione *S*-transferase
IP: immunoprecipitation
MAP1LC3B/LC3B: microtubule-associated protein 1 light chain 3 beta
PIK3C3: phosphatidylinositol 3-kinase, catalytic subunit type 3
SQSTM1: sequestosome 1
TMEM166: transmembrane protein 166
