# Supplementary material for: TMEM166/EVA1A interacts with ATG16L1 and induces autophagosome formation and cell death
Source: Cell Death Dis. 2016 Aug 4;7(8):e2323–. doi: 10.1038/cddis.2016.230 (PMC5108317; doi:10.1038/cddis.2016.230)
Supplement: Supplementary Table S1 [file cddis2016230x1.docx]

Table s1 Sense sequences of indicated siRNAs in this study

| siRNA | Targeting sequences |
| --- | --- |
| *EVA1A* siRNA |  |
| *EVA1A-1* | 5’-CUAGCGGCCUAUUCCUUUG-3’ |
| *EVA1A-2* | 5’-UGAUAAGGAUCUCUUGCCA-3’ |
| *EVA1A-3* | 5’-GAGCCUGAAUCGCUACUAU-3’ |
| *EVA1A-4* | 5’-AGAUCUGGAUGAAUGGCCA-3’ |
| *EVA1A-5* | 5’-GAGCAGCAGCGACAGCAGC-3’ |
| *VPS34* siRNA |  |
| *VPS34-1* | 5’-GUGUGAUGAUAAGGAAUAU-3’ |
| *VPS34-2* | 5’-GUUCUCAGGACUAUAUCAA -3’ |
| *ATG7* siRNA | 5’-CAGUGGAUCUAAAUCUCAAACUGAU -3’ |
| *ATG5* siRNA | 5’-GCAACUCUGGAUGGGAUUG-3’ |
| *BECN1* siRNA | 5’-GCUGCCGUUAUACUGUUCU-3’ |
| *ATG16L1* siRNA | 5’-CCCGUGAUGACUUGCUAAA-3’ |
| Control siRNA | 5’-UUCUCCGAACGUGUCACGU-3’ |
