# Supplementary material for: TMEM166/EVA1A interacts with ATG16L1 and induces autophagosome formation and cell death
Source: Cell Death Dis. 2016 Aug 4;7(8):e2323–. doi: 10.1038/cddis.2016.230 (PMC5108317; doi:10.1038/cddis.2016.230)
Supplement: Supplementary Materials and Figure Legends [file cddis2016230x2.docx]

**Supplementary data**

**TMEM166/EVA1A interacts with ATG16L1 and induces autophagosome formation and cell death**

Jia Hu^1,2,5^, Ge Li^1,5^, Liujing Qu^1^, Ning Li^3^, Wei Liu^3^, Dan xia^1^, Beiqi Hongdu^1^, Xin Lin^1^, Chentong Xu^1^, Yaxin Lou^4^, Qihua He^4^, Dalong Ma^1^, Yingyu Chen^1^*

^1^ Department of Immunology, Peking University School of Basic medical science; Key Laboratory of Medical Immunology, Ministry of Health, Peking University Health Sciences Center, 38 Xueyuan Road, Beijing, 100191, China.

^2^ Research center for Tissue Engineering and Regenerative Medicine. Wuhan Union Hospital, 1277 Jiefang Road, Wuhan, 430022, China

^3^ Department of Biochemistry and Molecular Biology, Program in Molecular and Cell Biology, Zhejiang University School of Medicine, Hangzhou, 310058, China

^4^ Medical and Healthy Analytical Center, Peking University, 38 Xueyuan Road, Beijing, 100191, China.

^5^These authors contributed equally to this work.

*Corresponding author: Yingyu Chen, Key Laboratory of Medical Immunology, Ministry of Health, Peking University Health Sciences Center, 38 Xueyuan Road, Beijing, 100191, China. Tel & Fax: +86-10-82801149; E-mail: yingyu_chen@bjmu.edu.cn

**Materials and Methods**

**The fluorescence protease protection (FPP) assay.** HEK293 cells were planted in the LabTek chambers (Nalge Nunc International) and transfected with constructs encoding the proteins CFP and YFP-EVA1A-Cherry or CFP and Cherry-EVA1A-YFP. The cells were washed three times for 1 min each in KHM buffer (110 mM potassium acetate, 20 mM HEPES, 2 mM MgCl2) at room temperature, then permeabilized with 20 µM of digitonin diluted in KHM buffer for 1min. After washing cells with KHM buffer, 4 mM of the protease trypsin diluted in KHM buffer was added directly to the cells and immediately start live cell imaging to record ﬂuorescent signals. Images were captured in multi-tracking mode on a laser scanning confocal microscope (LSM510 Meta, Carl Zeiss) with a 63 × Plan Apochromat 1.4 NA objective.

**Supplementary figure legends**

**Figure S1.** **Knockdown of *EVA1A* increases autophagosome formation**. **(a)** U2OS cells were transfected with the *siControl* or *siEVA1As* for 48 h. The levels of *EVA1A* mRNA were detected by Reverse transcription. (**b**) U2OS cells were transfected with *siControl* or *siEVA1A* for 48 h, then treated with EBSS for the last 2 h. Representative confocal microscopic images of endogenous LC3 distribution were shown. (**c**) Cell treatment was as same as (d), Quantification of LC3 puncta per cell were analyzed. Data are the mean ± SD of at least 100 cells scored. **P* < 0.05, ****P* < 0.001.

**Figure S2.** **EVA1A is a type I membrane protein and locates on cis-Golgi apparatus.** **(a)** HEK293 cells were cotransfected with EVA1A-MYC and P58-GFP-expressing plasmids for 24 h and then stained with an anti-MYC antibody. **(b)** HEK293 cells were transfected with EVA1A-MYC expressing plasmids for 24 h and then stained with an anti-MYC and anti-TGN46 antibody. Representative confocal microscopic images were shown. **(c and d)** HEK293 cells were transfected with CFP and YFP-TMEM166-Cherry **(c)** or CFP and Cherry-TMEM166-YFP **(d)** for 24 h, then permeabilized with digitonin and followed by trypsin treatment for indicated times. The ﬂuorescent signals were recorded by confocal microscope.

**Figure S3** **Combination of Ad5-EVA1A and autophagy-inducer increases cell death.** (**a, b, and c**) U2OS cells were infected with either Ad5-EVA1A or Ad5-null for 24 h before treated with or without EBSS **(a),** or RAPA (5 μm) **(b)** or CQ (25 μm) for 8 h. Cell death was measured by FITC–Annexin V plus PI staining followed by flow cytometry analysis.

**Figure S4. Colocalization of EVA1A mutant with ZFVYE1, LC3B, and ATG16L1.** (**a-c**)U2OS cells were cotransfected with FLAG-EVA1A_60-152_ and GFP-ZFVYE1 (**a**), GFP-LC3B (**b**), and GFP-ATG16L1 (**c**) expressing plasmids for 24 h and then stained with an anti-FLAG antibody. (**d-f**) U2OS cells were cotransfected with FLAG-EVA1A_30-152_ and GFP-ZFVYE1 (**d**), GFP-LC3B (**e**), GFP-ATG16L1 (**f**) expressing plasmids for 24 h and then stained with an anti-FLAG antibody. (**g-i**) U2OS cells were cotransfected with FLAG-EVA1A_1-60_ and GFP-ZFVYE1(**G**), GFP-LC3B (**h**), and GFP-ATG16L1 (**i**) expressing plasmids for 24 h and then stained with an anti-FLAG antibody.

**Figure S5. The correlation of EVA1A and ATG12‒ATG5/ATg16L complex. (a)** HeLa cells were cotransfected with GFP-ATG16L1, GFP-ATG5 and FLAG-EVA1A_60-152_ for 24 h, then incubated with EBSS for 2 h. Total cell extracts were subjected to IP using either an anti-FLAG or a nonspecific control mIgG. GFP and FLAG were detected in the washed beads by Western blot. **(b)** GST-EVA1A_60-152_ fusion protein and the GST protein immobilized on Glutathione-Sepharose beads were incubated with GFP-ATG16L1 transfected HeLa cell lysates at 4 ℃ for 4 h. GFP, ATG12-ATG5 and GST were detected in the washed beads by western blot.
