# Supplementary figures and images for: TMEM166/EVA1A interacts with ATG16L1 and induces autophagosome formation and cell death
Source: Cell Death Dis. 2016 Aug 4;7(8):e2323–. doi: 10.1038/cddis.2016.230 (PMC5108317; doi:10.1038/cddis.2016.230)

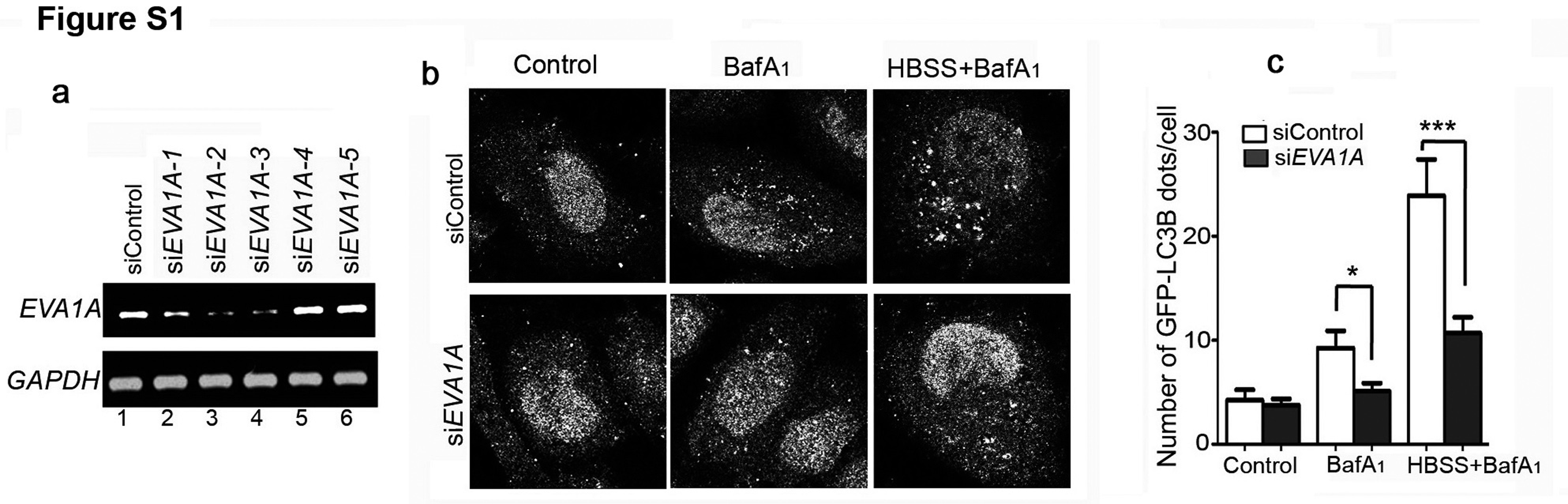

Supplement: Supplementary Figure S1 [file cddis2016230x3.tif]

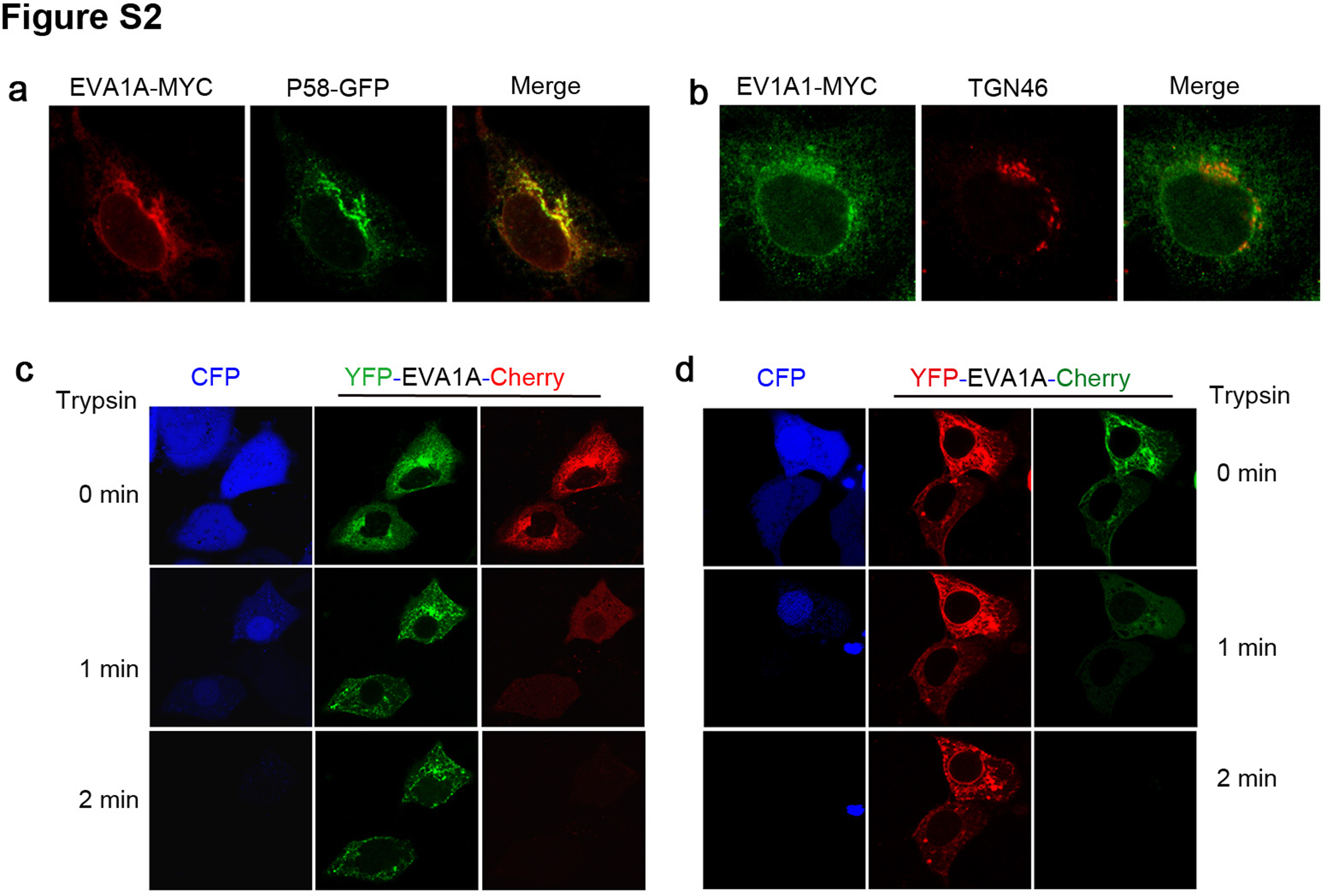

Supplement: Supplementary Figure S2 [file cddis2016230x4.tif]

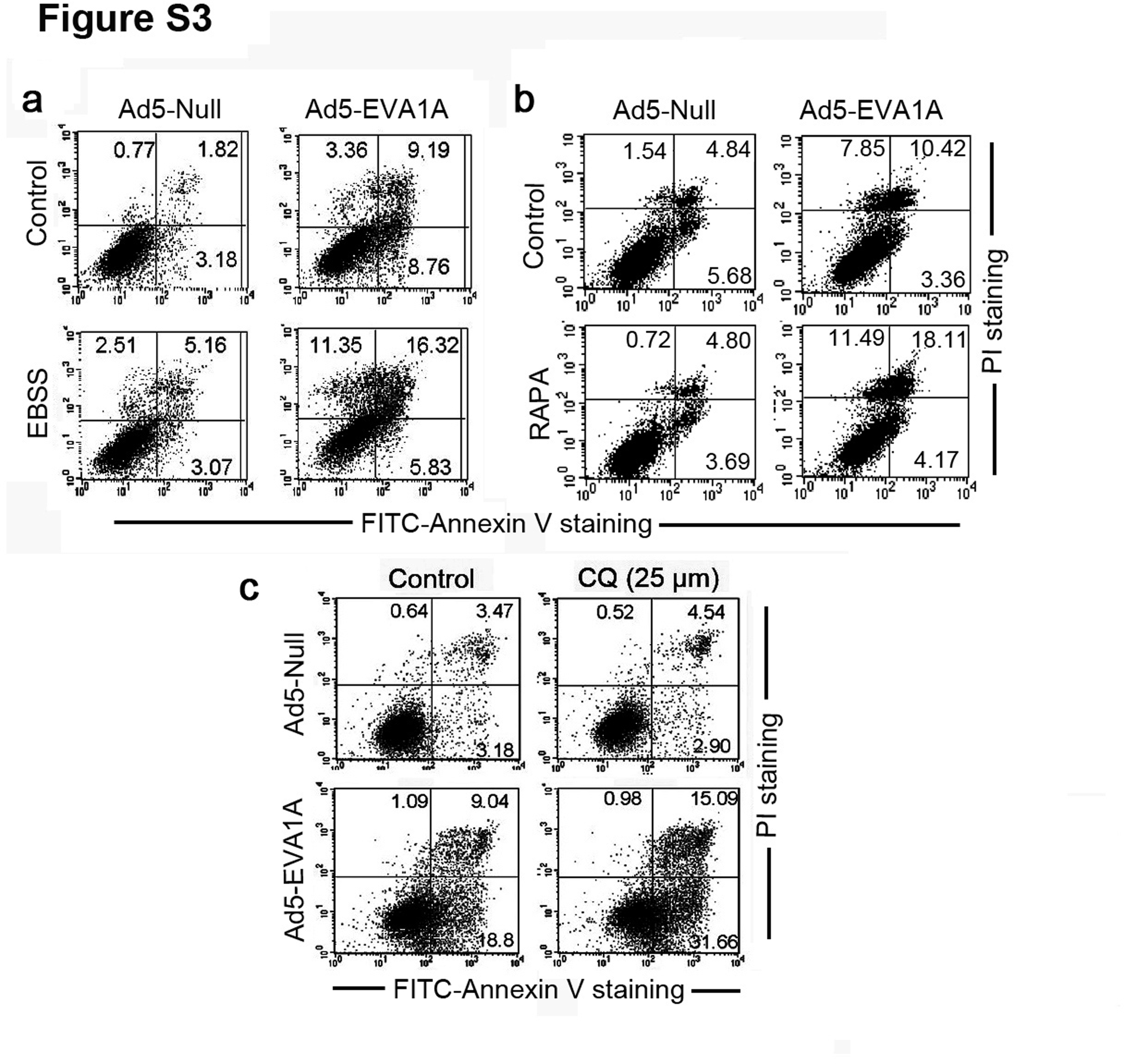

Supplement: Supplementary Figure S3 [file cddis2016230x5.tif]

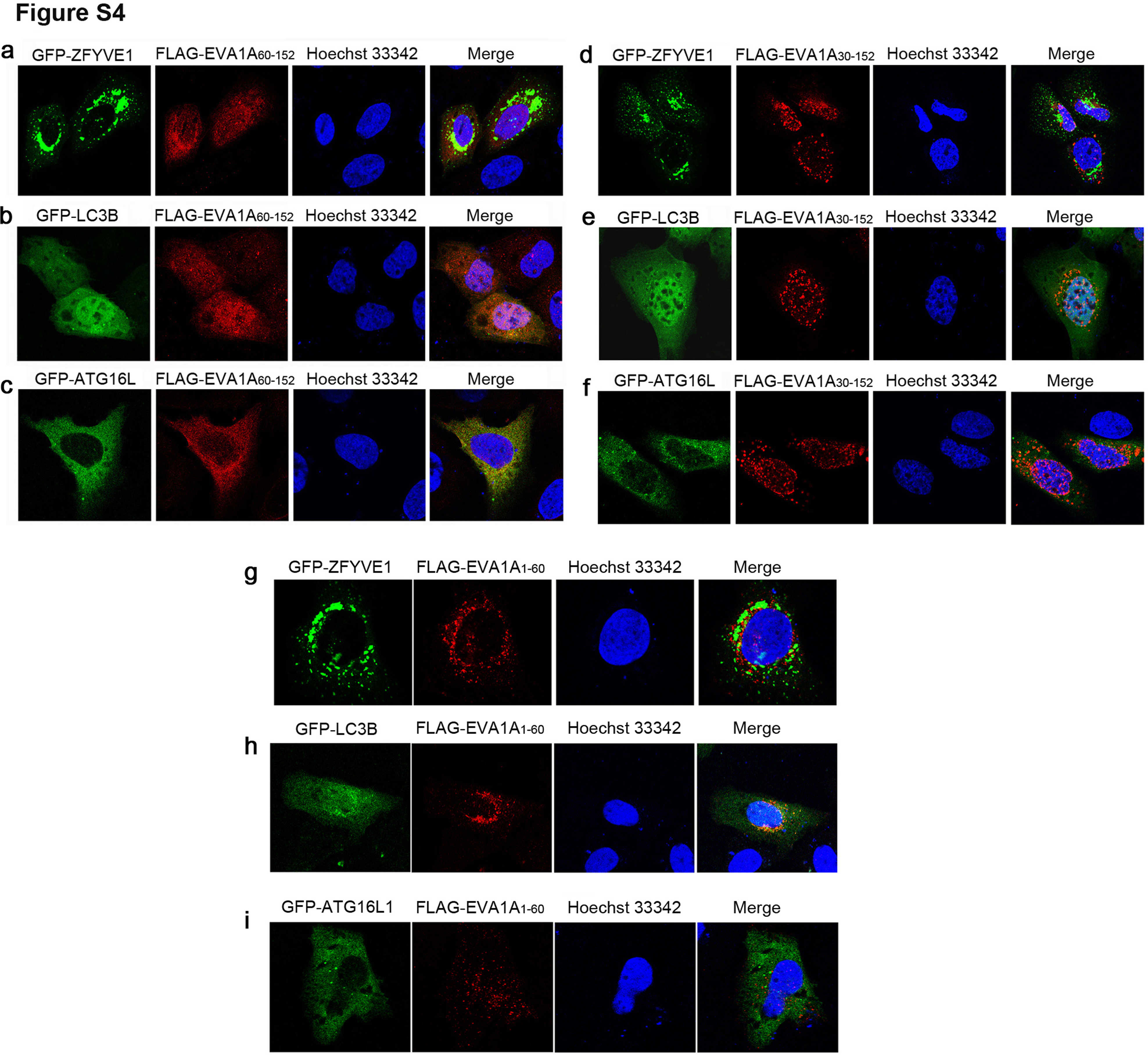

Supplement: Supplementary Figure S4 [file cddis2016230x6.tif]

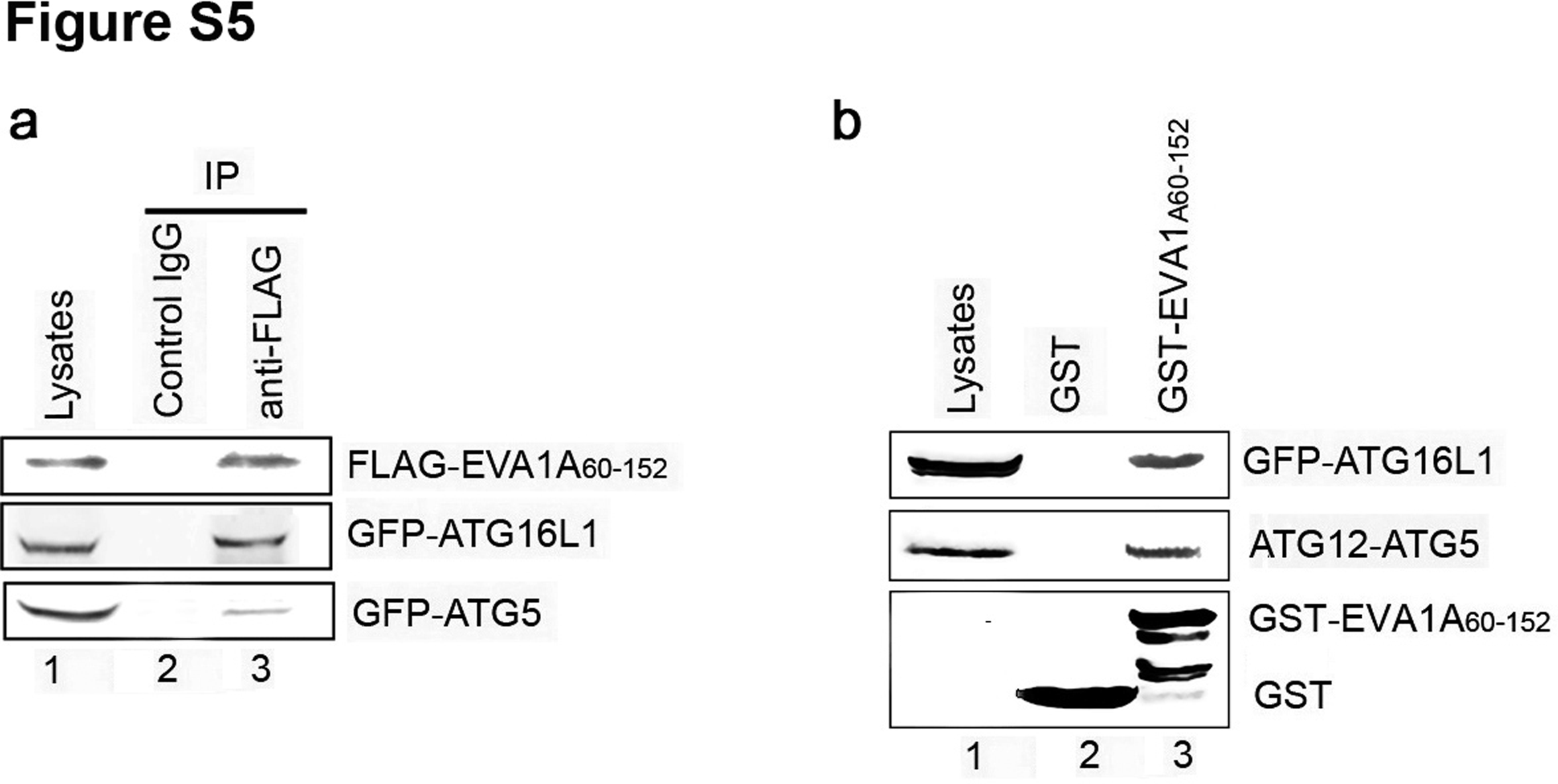

Supplement: Supplementary Figure S5 [file cddis2016230x7.tif]
